# Advancements in fertility preservation strategies for pediatric male cancer patients: a review of cryopreservation and transplantation of immature testicular tissue

**DOI:** 10.1186/s12958-024-01219-5

**Published:** 2024-04-18

**Authors:** Zih-Yi Sung, Yong-Qi Liao, Jung-Hsiu Hou, Hong-Hsien Lai, Sung-Ming Weng, Hai-Wei Jao, Buo-Jia Lu, Chi-Huang Chen

**Affiliations:** 1 School of Medicine, College of Medicine, Taipei Medical University, Taipei, Taiwan, ROC; 2 Graduate Institute of Medical Science, College of Medicine, Taipei Medical University, Taipei, Taiwan, ROC; 3 Division of Reproductive Medicine, Department of Obstetrics and Gynecology, Taipei Medical University Hospital, Taipei, Taiwan, ROC; 4 Department of Obstetrics and Gynecology, School of Medicine, College of Medicine, Taipei Medical University, Taipei, Taiwan, ROC

**Keywords:** Immature testicular tissue, Cryopreservation, Vitrification, Slow freezing, Cryoprotectant, Tissue engineering, Scaffold

## Abstract

Recently, there has been increasing emphasis on the gonadotoxic effects of cancer therapy in prepubertal boys. As advances in oncology treatments continue to enhance survival rates for prepubertal boys, the need for preserving their functional testicular tissue for future reproduction becomes increasingly vital. Therefore, we explore cutting-edge strategies in fertility preservation, focusing on the cryopreservation and transplantation of immature testicular tissue as a promising avenue. The evolution of cryopreservation techniques, from controlled slow freezing to more recent advancements in vitrification, with an assessment of their strengths and limitations was exhibited. Detailed analysis of cryoprotectants, exposure times, and protocols underscores their impact on immature testicular tissue viability. In transplantation strategy, studies have revealed that the scrotal site may be the preferred location for immature testicular tissue grafting in both autotransplantation and xenotransplantation scenarios. Moreover, the use of biomaterial scaffolds during graft transplantation has shown promise in enhancing graft survival and stimulating spermatogenesis in immature testicular tissue over time. This comprehensive review provides a holistic approach to optimize the preservation strategy of human immature testicular tissue in the future.

## Background

Recently, there has been increasing emphasis on the gonadotoxic effects of cancer therapy in prepubertal boys [1]. Both chemotherapy and radiotherapy have the potential to damage immature testicular tissue (ITT) [2, 3]. In a study assessing the fertility potential of 44 boys who underwent combination chemotherapy for acute lymphoblastic leukemia, it was found that their fertility potential was only 50% compared to their age-matched peers. Notably, 18 of these boys exhibited significantly reduced fertility [4]. This damage to the testicular tissue can result in future infertility [5]. However, with significant advancements in the oncological treatment of childhood cancer, the survival rates have reached up to 80% [6, 7]. As a result, a majority of these boys are expected to become long-term survivors of childhood malignancies and will eventually require their intact and functional testicular tissue for reproduction.

Male fertility preservation in prepubertal boys has been a topic of discussion for an extended period. Various approaches have been explored, including cryopreservation of spermatogonial stem cells (SSCs) [8], in vitro spermatogenesis using different cell culture systems such as three-dimensional (3D) culture systems [9], and cryopreservation of ITTs followed by transplantation. Although there have been no reported outcomes regarding clinical autotransplantation of human ITT, several animal studies have achieved successful restoration of spermatogenesis and even the production of offspring [10–13]. A significant breakthrough in ITT preservation was achieved in 2019 when cryopreserved ITT autografts successfully resulted in offspring in a study conducted on non-human primates (NHPs) [11]. Hence, ITT cryopreservation and transplantation are regarded as the most promising methods.

Cryopreservation techniques, such as slow freezing or vitrification, have been employed for preserving human ITT, with the aim of maintaining SSCs for future autologous transplantation or in vitro maturation during adulthood [14]. To optimize the preservation of ITT, a thorough comparison between vitrification and CSF techniques was conducted in this study. Several studies have also suggested that vitrification with low-concentration cryoprotectants results in minimal damage to ITT and reduces the likelihood of harm [15]. Furthermore, various transplantation strategies exist for ITT transplantation. In this study, we analyze the merits and demerits encountered during the transplantation procedure, along with the optimal graft size and transplantation site considerations for both autologous and xenologous transplantation approaches.

In addition to the background issues, several add-on materials or techniques have been introduced to improve the outcomes of cryopreservation and transplantation. Tissue engineering scaffolds serve the purpose of modulating the cellular microenvironment, enabling precise control over physical, chemical, and biological factors. By creating environments that promote proper cellular differentiation within transplanted ITT, these scaffolds can effectively address the compromised capacity for cellular differentiation observed upon thawing and transplantation. These scaffolds not only provide structural support but also demonstrate biocompatibility and biodegradability [16]. Besides, stem cell therapeutic drugs may also be added to support cell viability and restore testicular tissue functionality upon transplantation [17]. Nanotechnology, including encapsulation in hydrogels, has been employed to facilitate the delivery of essential nutrients and cell-secreted molecules. Optimal nutrient and oxygen supply can create a more favorable environment for the restoration of testicular tissues following ITT grafting [18]. However, because human live birth following ITT transplantation has not yet been achieved, many studies continue to explore enhanced cryopreservation, transplantation strategies and scaffolds in tissue engineering within this domain. Therefore, this review aims to offer an overview of the current knowledge and provide updated insights into preserving ITT. Our emphasis has been on systematically discussing the categorized preservation and transplantation of ITT across three primary components: cryopreservation strategies, transplantation strategies, and the biomaterial scaffolds applied for transplantation.

## Cryopreservation strategies

The process of acquiring testicular tissue is straightforward. Using a mouse as an example, the procedure starts with administering anesthesia and performing disinfection. This is followed by a small abdominal incision to reveal the testis. Careful isolation of the testicular tissue is then carried out, ensuring minimal harm to adjacent structures. The collected tissue is then placed in an appropriate container for further steps, like cryopreservation.

Although cryopreservation techniques for ITT in humans are still experimental, they are generally regarded as acceptable since they allow for the survival and proliferation of spermatogonia [19, 20]. However, studies have observed the loss of germ cells and impaired differentiation following the xenografting of cryopreserved and fresh human ITT into a mouse model [21–23].

Human samples are limited since there are many ethical issues that should be considered. As a result, we also include research from other species, such as NHPs, rats, and piglets, to discuss the optimization of cellular preservation techniques.

### Controlled slow freezing (CSF)

CSF involves the deliberate and gradual cooling of ITT, allowing for the preservation of germ cells and SSCs that possess the capacity to differentiate and develop into functional spermatozoa. By carefully controlling the freezing process, optimal conditions are maintained to ensure the viability and future functionality of the preserved tissue. This technique offers several advantages, including the potential for long-term storage, indefinite preservation of the ITT, and the ability to restore fertility in the future through techniques such as testicular tissue transplantation or in vitro maturation.

CSF has been proven to be a feasible cryopreservation method for ITT in several animal studies. Abrishami et al. performed a study involving the xenografting of immature porcine testicular tissues subjected to slow freezing. The study revealed the presence of spermatids in recipient mice after orthotopic xenografting for 6 months [24]. Moreover, in comparison to in vivo transplantation, mouse ITT subjected to slow freezing exhibited a higher likelihood of achieving in vitro maturation [25–28]. In addition to spermatogenesis, successful offspring production was observed in recipient mice after transplantation of ITT using CSF. In 2002, Shinohara et al. demonstrated the successful birth of mouse offspring through in-vitro microinsemination, and rabbit offspring were obtained using rabbit sperm that were developed in fresh transplants in a xenogeneic surrogate mouse [10]. In 2019, Fayomi et al. found that complete spermatogenesis was confirmed in all thawed ITT grafts after CSF, and the first NHP offspring was safely produced after autograft implantation [11]. In these cases, CSF was employed to preserve testicular pieces, highlighting its effectiveness in preserving and utilizing reproductive cells for successful reproduction [10, 11]. Due to these remarkable outcomes, in subsequent years, numerous studies have been published, with a primary focus on utilizing recipient mice for ITT grafts following CSF [29, 30].

Nevertheless, it is important to highlight that the slow freezing method did not achieve full spermatogenesis and offspring production in human tissue [31]. Wyns et al. discovered that slow freezing can preserve the proliferation capacity of spermatogonial cells even after 6 months of xenografting, which may have beneficial effects on the long-term survival of the tissue. However, despite these positive effects, the tissues were unable to complete spermatogenesis [23]. In a study conducted by Rives-Feraille et al. in 2021, they cryopreserved and subsequently stored ITT obtained from prepubertal boys with cancer by CSF. Their findings indicated that there was no significant difference in seminiferous tubule integrity between fresh tissue and frozen-thawed tissue [31].

In summary, spermatids were observed in animal ITT grafts after being frozen using CSF. In human ITT, the preservation of tissue integrity may be observed following CSF cryopreservation, while the capacity for cellular differentiation may be compromised upon thawing and transplantation in several species like mice, NHPs and others according to our references. Consequently, to achieve successful spermatogenesis or offspring production via the transplantation of cryopreserved ITT, the implementation of supplementary elements such as tissue engineering methodologies or cryoprotectants becomes imperative.

### Vitrification

Vitrification is a technique that involves ultrarapid cooling of tissue or cells to extremely low temperatures, thereby bypassing the formation of ice crystals and preserving delicate cellular structures. This method has garnered significant attention in the field of female fertility preservation, particularly for oocytes and embryos. In recent years, researchers and clinicians have started exploring the application of vitrification to ITT, aiming to preserve the germ cells and SSCs that hold intrinsic reproductive potential within the testes. There are various protocols to apply vitrification, such as solid surface vitrification (SSV), open-pulled straw (OPS) vitrification and glass micropipette (GMP) vitrification. Several animal and human studies have recently used vitrification to preserve ITT with promising results.

Evaluating tissue morphology is a crucial aspect in assessing the viability of vitrification techniques. Poels et al. discovered that vitrified ITT from NHPs, when grafted onto nude mice, exhibited negligible histologic damage compared to fresh tissue [32]. Additionally, their research revealed that vitrification maintained the proliferation capacity of human spermatogonia in the following year [14]. Radaelli et al. also reported the successful preservation of morphological integrity in human ITT using the vitrification protocol [33].

**Fig. 1:**
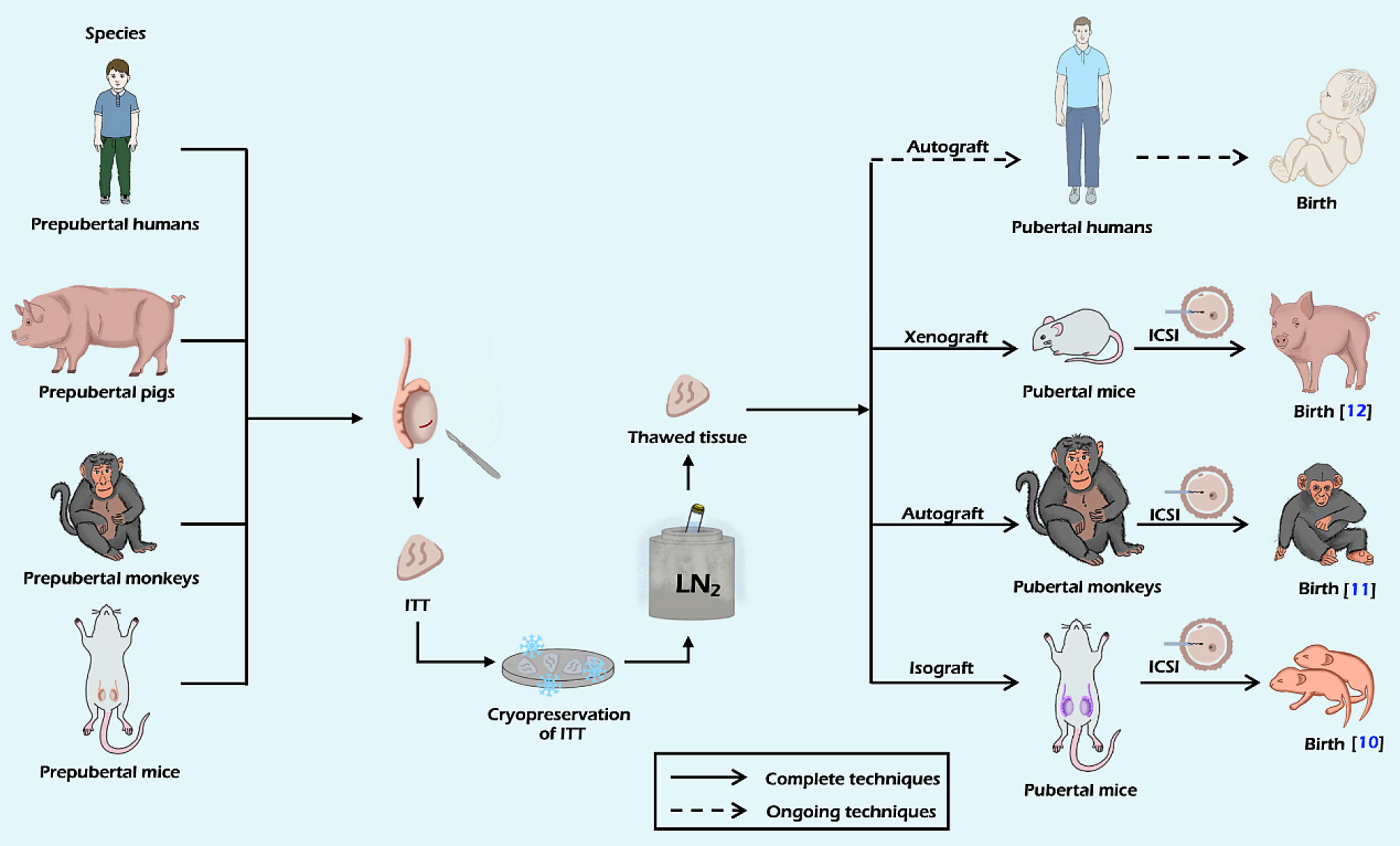
Schematic overview of fertility preservation through cryopreserved, thawed and transplanted ITT. The ITT could be cryopreserved by vitrification or CSF, and then isografted, autografted, or xenografted into species after being thawed. Here were the major milestones in offspring production following these cryopreservation methods. Offspring production was both observed through CSF and vitrification. Researches in this area are currently limited to animal models. LN_2_, liquid nitrogen

Spermatogenesis can occur after the process of vitrification. Lu et al. demonstrated the presence of spermatogenesis in vitrified ITT of mice with the assistance of scaffolds [13]. Yamini et al. found that the potential for spermatogenesis development in the vitrified graft was comparable to that of the fresh graft [34]. Additionally, successful offspring production has been observed following grafts involving cryopreserved tissues (Fig. 1). Kaneko et al. demonstrated this by obtaining sperm from xenografted piglet testicular tissue, resulting in the successful generation of offspring [12].

### Comparison

Preserving ITT lacks a universally standardized protocol. To optimize the preservation of ITT, a thorough comparison between vitrification and CSF techniques is conducted. Various indicators are considered to evaluate the feasibility and functionality of each cryopreservation method, including tissue survival, tissue morphology, spermatogenesis, and even the successful production of offspring. These parameters provide a comprehensive assessment of the efficacy and potential of each cryopreservation approach.

Vitrification is a relatively novel and cost-effective cryopreservation method that has demonstrated comparable or even superior effectiveness compared to CSF. Recently, several studies have been conducted to demonstrate its feasibility by observing the morphology of ITT. Curaba et al. found that germ cells and Sertoli cells exhibited comparable levels of preservation following vitrification, akin to those observed in the fresh and slow-freezing control groups [35]. Furthermore, Poels et al. conducted a study comparing xenotransplantation of fresh, frozen-thawed, and vitrified-warmed human ITT into nude mice, and found that the spermatogonia recovery rate, encompassing both survival and proliferative capacity, demonstrated a swifter pace in the vitrification group compared to the CSF group. These findings suggest that vitrification may offer advantages in terms of spermatogonial recovery compared to CSF techniques [14].

The most suitable cryopreservation methods for ITT tissue may differ between species. Shinohara et al. demonstrated mouse offspring production via ITT cryopreservation using CSF followed by grafting [10]. In xenografts of sheep ITT, Pukazhenthi et al. found that CSF was better than vitrification for preserving the function and integrity of the tissue, indicating its superiority in maintaining the reproductive potential of sheep ITT [36]. In addition, Fayomi et al. reported the successful production of NHP offspring through CSF and subsequent autografting of ITT [11]. In contrast, Kaneko et al. substantiated the achievement of viable piglet reproduction subsequent to xenografting of vitrified ITT. These findings indicate the successful application of vitrification and CSF in preserving and utilizing ITT for reproductive purposes. Nevertheless, these outcomes highlight the potential for the development of species-specific cryopreservation techniques. Further research is necessary to understand these variations and determine the optimal cryopreservation method for each species.

In summary, the outcomes of ITT cryopreservation varied across different methods and even different species. Both CSF and vitrification are feasible cryopreservation methods for ITT according to the current evidences. A brief summary of cryopreservation methods is shown in Table 1. Numerous studies are still pursuing more ideal cryopreservation methodologies. In the case of human beings, evidence-based studies are still limited, making it challenging to determine the most suitable method. Therefore, a significant trajectory lies ahead in the continued advancement of research within this domain.

**Table 1:** Studies of ITT cryopreservation through CSF and vitrification

	Reference	ITT Samples	Experimental design	Outcome
CSF	Shinohara et al. [10] 2002	Mice or rabbits	Xenograft/isograft (to mice)	Offspring production occurred in both mouse and rabbit testicular pieces
	Wyns et al. [22] 2007	Human	Xenograft (to mice)	The survival and proliferation of spermatogonia and Sertoli cells were observed
	Wyns et al. [23] 2008	Human	Xenograft (to mice)	Spermatogonia could survive and proliferate but could not complete the spermatogenesis
	Abrishami et al. [54] 2010	Piglets	Xenograft (to mice)	Tissue fragments exhibited spermatogenesis when cryopreserved with glycerol
	Fayomi et al. [11] 2019	Rhesus macaques (NHP)	Autograft	The first offspring of primate was produced
	Rives-Feraille et al. [31] 2022	Human	In vitro observation after thawing	The ITT structure remained intact, and DNA expression resembled that of fresh tissue
Vitrification	Poels et al. [32] 2012	Macaca mulatta (NHP)	Xenograft (to mice)	The structural integrity and function of tissue was comparable to fresh one
	Kaneko et al. [12] 2013	Piglets	Xenograft (to mice)	Mature sperm were seen and the offspring of piglets were produced
	Yamini et al. [34] 2016	Mice	Allograft	The recovery rate and germ cell differentiation of the graft were similar to the fresh tissue
	Radaelli et al. [33] 2017	Mice	In vitro observation after thawing	The tissue exhibited intact morphology and minimal cryodamage with reduced cryoprotectant exposure
	Lu et al. [13] 2022	Mice	Isograft	Spermatogenesis was observed, and tissue integrity resembled that of fresh tissue
Comparison	Curaba et al. [35] 2011	Human	In vitro culture	SSV and CSF had similar success rates for preserving tissue, based on morphology
	Poels et al. [14] 2013	Human	Xenograft (to mice)	The recovery rate of spermatogonia was faster in vitrification
	Pukazhenthi et al. [36] 2015	Lambs	Xenograft (to mice)	CSF was significantly better in preserving cellular integrity and function

Both cryopreservation methods had similar outcomes in spermatogenesis and tissue integrity after thawing. Offspring production occurred both in vitrification and CSF. ITT, immature testicular tissue; CSF, controlled slow freezing; SSV, solid-surface vitrification; NHP, non-human primate

### Cryoprotective agent (CPA)

The choice of cryopreservation protocol depends on various factors, such as the type of tissue being banked, the intended use of the tissue, and the available resources. It is important to note that cryopreservation can have negative effects on tissue viability and function, and optimization of the protocol is necessary to minimize these effects [37]. During the process of reaching temperatures down to -196 °c, there might be some damage to ITT — like crystal formation, which causes injury to tissues and cells. CPAs are substances used to protect cells and tissues during the freezing process. Commonly utilized CPAs in cryopreservation include glycerol, dimethyl sulfoxide (DMSO), ethylene glycol (EG), and 1,2 - Propanediol (PrOH).

DMSO is the most frequently used CPA in the cryopreservation of ITT. It functions by permeabilizing the cell membrane and interacting with hydrogen molecules, effectively reducing the formation of ice crystals within the cells during the cryopreservation process [38]. In 2022, Lu et al. discovered that vitrified ITT with 10.6% (approximately 1.4 M) DMSO exhibited larger seminiferous tubules than vitrified ITT without CPA. These findings indicated that adding DMSO as a CPA during vitrification protected the tissue integrity [13].

EG is another commonly used CPA due to its ability to lower the freezing point of water, making it useful in cryopreservation. It can penetrate the cells, replace water molecules and lower the water freezing point, helping to prevent ice crystal formation and maintain the integrity of the biological material during freezing and subsequent thawing [39]. Several studies compared its effectiveness with DMSO, and found that the ideal CPA may differ in accordance with the developmental stage of the tissue. Unni et al. conducted a study using testicular tissue from both immature and adult rats, as well as adult humans, to assess the impact of CPA on tissue of different ages. For ITT, DMSO was found to be an effective CPA, whereas EG was more suitable for adult testicular tissue [40]. However, the study did not include human ITT as a comparison, leaving uncertainty regarding the most appropriate CPA for human ITT cryopreservation. Goossens et al. compared 1.5 M EG and 1.5 M DMSO to evaluate their effects on ITT from mice after CSF. Although spermatozoa were observed in both the DMSO and EG groups, DMSO grafts grew larger, and had less tubular damage, including fewer detached cells and fluid accumulation [41]. Furthermore, Jahnukainen et al. discovered that DMSO outperformed EG as a cryoprotectant while using ITT from NHP in xenografts. 30% of the grafts survived followed by cryopreservation with 1.4 M DMSO, whereas grafts cryopreserved with EG only yielded a 7% recovery rate [42]. In conclusion, DMSO demonstrated superior cryoprotective properties compared to EG for preserving ITT.

Moreover, numerous studies have compared DMSO with other CPAs. Milazzo et al. found that 1.5 M DMSO had a more favorable outcome in maintaining architecture, viability, endocrine, and partial exocrine functions in mice ITT after CSF compared to 1.5 M PrOH [43]. Similarly, Keros et al. revealed that 0.7 M DMSO demonstrated superior preservation of the structure of human ITT, particularly spermatogonia, when compared to PrOH or glycerol in CSF. This protocol has been widely adopted for cryopreservation in various research studies [14, 22, 23, 35]. Furthermore, Fayomi et al. also utilized the same DMSO-based protocol for preserving ITT from NHPs through CSF, leading to successful offspring production after autografting [11]. These collective findings suggest that DMSO is a promising and effective CPA for preserving ITT.

Recently, a mixture of different CPAs has been studied to optimize the preservation of ITT in vitrification. The protocol derived from Abrishami et al. indicated that the two most effective cryopreservation methods, programmed slow-freezing with glycerol and vitrification with a 5-minute exposure to a mixture of 15% DMSO and 15% EG (DMSO-VS2 group), exhibited similar post-thawing cell survival rates [24]. Five minutes of DMSO-VS2 exposure resulted in larger tubule diameters than 15 min of exposure. In addition, no grafts were recovered from the 30-minute exposure group, indicating that long exposure to high concentrations of CPA may have toxic effects on the testis tissue. There was also a widely used protocol (7.5% EG and 7.5% DMSO) in vitrification [14, 15, 32, 33]. The protocol was originally introduced by Poels et al. [32] and it was slightly modified from the study conducted by Abrishami et al. mentioned above [24]. Briefly, testicular tissue from NHPs was pretreated with an equilibration solution of 7.5% EG and 7.5% DMSO for 10 min at 4 °C. Then, it was transferred to the vitrification solution consisting of 15% EG and 15% DMSO for 5 min at 4 °C. The survival of proliferative spermatogonia and functional Leydig cells was confirmed in both fresh and vitrified grafts, suggesting that vitrification with a mixed CPA protocol is a promising way to preserve these cell types.

Additionally, several studies have explored the use of additives in combination with CPAs. Some common additives include trehalose, glycerol, sucrose, hypotaurine and melatonin. The incorporation of specific additives with CPAs has shown the potential to enhance cell viability and functionality after cryopreservation. By mitigating the cellular damage during freezing and thawing, this combination improves the chances of successful recovery and growth of the preserved cells and tissues. For instance, Jung et al. evaluated whether additives (trehalose, hypotaurine, necrostatin-1 or melatonin) could improve the performance of DMSO during primate ITT cryopreservation by CSF [44]. The study revealed that slow freezing with 1.4 M DMSO and 200 mmol/L trehalose emerged as an effective CPA for preserving the tissue based on the survival rate and number of recovered cells. These findings highlight the potential benefits of incorporating specific additives with CPAs.

Currently, a universally accepted standard protocol has yet to be established. Most of the mentioned studies were conducted among animals; therefore, the establishment of an optimal cryopreservation protocol for human ITT banking remains an active area of research. However, it is anticipated that human ITT will increasingly serve as a valuable model for achieving desired outcomes such as spermatogenesis or successful offspring production in the future.

## Transplantation strategies

### Autologous transplantation

Autologous ITT transplantation is one of the mature techniques to produce sperm and even give birth to healthy offspring. This technique has produced successful spermatogenesis in rodents and also in higher primates [11, 45–47]. A summary of autologous testicular tissue grafting in NHPs is provided in Table 2.

**Table 2:** **Summary of autologous testicular tissue grafting in NHPs**

Reference	Testicular Tissue Donor	Grafting Size	Grafting Site	Cryopreserved or Fresh	Outcome
Wistuba et al. [45] 2006	4-week-old marmosets	0.5 to 1 mm^3^	Back skin	Fresh	Graft survival: 31.25%. Spermatogenesis was blocked at early meiosis
Luetjen et al. [46] 2008	Prepubertal and adult marmosets	0.5 to 1 mm^3^	(1) Back skin	Frozen/Thawed and fresh	Graft survival: 55.7%
			(2) Scrotal skin		Grafts in the scrotum exhibited complete spermatogenesis
Jahnukainen et al. [47] 2012	Pubertal rhesus monkeys	1 mm^3^	(1) Scrotal	Frozen/Thawed	Graft survival: 7% from scrotal, 4% from shoulder and 3% from arm
			(2) Shoulder		Spermatozoa was detected in the scrotal group
			(3) Arm		
Fayomi et al. [11] 2019	Prepubertal rhesus monkeys	9 to 20 mm^3^	(1) Back skin	Frozen/Thawed and fresh	Graft survival: 100%. All grafts exhibited complete spermatogenesis and produced a healthy baby
			(2) Scrotal skin		

It demonstrates enhanced graft survival when autologous testicular tissue grafting is performed in NHPs using larger graft sizes ranging from 9 to 20 mm^3^

As mentioned in Table 2, larger ITT fragments might lead to better tissue survival after transplantation. Nevertheless, a definitive cutoff value to determine the distinction between large and small grafts remains elusive. While smaller grafts, typically with a size smaller than 1mm^3^, may have better blood supply and a greater propensity to develop new blood vessels, facilitating their integration and survival at the recipient site, the outcomes of autografting were found to be unsatisfactory. There might be a certain risk of graft resorption when using smaller ITT fragments (1 mm^3^) for autografting [45–47]. In the study by Jahnukainen et al. [47], up to 95% of total cryopreserved grafts were lost due to graft resorption. Other studies also showed a poor survival rate of small-size grafts [45, 46]. On the other hand, Fayomi et al. [11]. employed fresh and frozen-thawed ITT fragments of larger sizes (ranging from 9 to 20 mm^3^) in castrated hosts, resulting in a 100% graft survival rate. The authors hypothesized that the improved outcomes of larger ITT fragments could be attributed to the graft’s ability to generate autocrine/paracrine factors, thereby overcoming ischemic injury. To our best knowledge, in studies related to autologous testicular tissue grafting in NHPs, only this study employed large fragments. Further studies utilizing larger ITT fragments may be necessary to provide additional evidence supporting their superiority over smaller ITT fragments.

Furthermore, the transplantation site is also the key factor that contributes to the success of graft survival. Autotransplantation of testicular tissue in higher primates has been performed at several sites, such as the back skin [11, 45, 46], the scrotal skin [11, 46, 47], the shoulder and the arm [47]. Spermatogenesis was blocked at the spermatocyte stage when grafting occurred in the ectopic site. In contrast, complete spermatogenesis was observed in the scrotal area. One benefit of transplanting to the scrotal site is its more stable temperature compared to ectopic sites. The scrotum’s temperature is closer to the body’s core temperature than peripheral areas like the skin or back. Additionally, in certain species, the scrotum contains muscles that, through the cremasteric reflex, help maintain a cooler temperature within the scrotum. This cooler environment is crucial for protecting the integrity of sperm-producing cells [48]. However, in a study by Fayomi et al. [11] in 2019, all grafts in the back and scrotal skin exhibited complete spermatogenesis. The method employed in this study involved suturing each testicular tissue fragment to the subcutaneous layer of the skin, rather than depositing as a slurry of small pieces in the subcutaneous space. The injury caused by the suture needles or skin flap incision appeared to be adequate in stimulating angiogenic granulation and vascularization of the apposed testicular tissue grafts, ultimately resulting in 100% graft survival and complete spermatogenesis. In conclusion, the scrotal site could become the favored option of graft location. Considering transplantation to an ectopic area may be viable, but it would require a more well-planned and delicate approach.

### Xenologous transplantation

Xenotransplantation of ITT is another technique in which scientists transplant ITT from one species to another. It may have certain disadvantages such as graft rejection, increased risk of zoonotic infections, and potential impairment in producing functional sperm or supporting regular testicular function in the long run. It is important to note that while xenotransplantation of ITT has several disadvantages, it allows for studying testicular development, maturation, and spermatogenesis across species. This technique can deepen our understanding of reproductive biology and contribute to advancements in fertility treatments. For instance, lots of mammalian species (e.g., mouse, rabbit, pig, goat, kitten, horse, cattle, sheep, dog, buffalo, NHPs, etc.) have been reported to complete spermatogenesis by xenografting techniques [10, 49–56]. A summary of xenologous testicular tissue grafting in mammalian species is provided in Table 3.

**Table 3:** Summary of xenologous testicular tissue grafting in mammalian species

Reference	Testicular Tissue Donor	Testicular Tissue Recipient	Grafting Size	Grafting Site	Cryopreserved or Fresh	Outcome
Shinohara et al. [10] 2002	Prepubertal Rabbits	ICR nude mice	8 to 16 mm^3^	Testicular parenchyma	Frozen/Thawed and fresh	Spermatogenesis occurrence: 74% in fresh and 90% in frozen transplants
						One male offspring was produced
Honaramooz et al. [49] 2002	Prepubertal ICR or B6C3F1 mice, pigs and goats	Immunodeficient mice	0.5 to 1 mm^3^	Back skin	Frozen/Thawed and fresh	Graft recovery: more than 60%
						Sperm were all viable and functional from testis grafts of all three species into mice
Snedaker et al. [50] 2004	Prepubertal domestic kittens	Immunodeficient mice	0.5 to 1 mm^3^	Back skin	Frozen/Thawed	Graft recovery: 45.5%
						Complete spermatogenesis occurred after transplantation
Rathi et al. [97] 2005	Prepubertal cattle	Immunodeficient mice	1 to 2 mm in diameter	Back skin	Fresh	Graft recovery: 80%
						In most grafts, spermatogenesis was arrested at the pachytene spermatocyte stage
Rathi et al. [52] 2006	Prepubertal, pubertal and adult horses	Immunodeficient mice	1 to 2 mm in diameter	Back skin	Fresh	Graft recovery: 65%
						The pubertal group leaded to the most complete spermatogenesis
Arregui et al. [53] 2008	Prepubertal sheep	Immunodeficient mice	1 mm^3^	Back skin	Fresh	Graft recovery: 95.7%
						Complete spermatogenesis occurred after transplantation
Abrishami et al. [24] 2010	Prepubertal, pubertal and adult dogs	Immunodeficient mice	2 mm^3^	Back skin	Fresh	Graft recovery: 79.2%
						Prepubertal and pubertal testis xenografts maintained complete spermatogenesis
Reddy et al. [55] 2012	Prepubertal buffalos	Immunodeficient mice	Weighing 8-10 mg	Back skin	Fresh	Graft recovery: 89.4% after 12 weeks and 78.3% after 24 weeks
						Complete spermatogenesis occurred only in intact recipients
Ntemou et al. [56] 2019	Prepubertal marmosets	Swiss Nu/Nu mice	0.8 to 1 mm^3^	(1) Back skin	Fresh	Graft recovery after 4 months: 21% from back skin and 50% from intratesticular
				(2) Intratesticular		

This highlights the frequent use of the back skin of immunodeficient mice as a graft site. ICR, Institute of Cancer Research

From xenografting experiments in mammalian species, we learned that many parameters may have a strong influence on graft recovery and functionality. As mentioned above, larger fragments might lead to better results. However, most of the xenografting studies in non-human mammal species used graft sizes smaller than 2 mm^3^. To the best of our knowledge, larger graft sizes have been infrequently employed in xenotransplantation studies, making it uncertain whether smaller grafts would exhibit superior survival rates. In fact, the specific survival rates of small versus large grafts can vary depending on factors such as graft type, grafting technique, and individual characteristics. Therefore, it is important to determine an optimal tissue size for every species to ensure successful outcomes.

As mentioned in Table 3, the back skin of immunodeficient mice is commonly used for grafting ITT. This approach has the advantage of being minimally invasive. Alternatively, grafting the tissue into the testicular parenchyma or the scrotum may provide a more natural environment for the development of sperm, but these kinds of approaches may be more invasive and may require a higher level of operational skills. A study by Ntemou et al. [56] in 2019 demonstrated that the intratesticular transplants led to a higher final graft recovery rate and all recovered transplants showed complete spermatogenesis. The authors hypothesized that the testis was a well vascularized organ that could prevent post-transplantation hypoxia and provide sufficient oxygen for transplant survival. The primary advantage of intratesticular grafting lies in its optimal temperature regulation and the unique hormonal environment within the testes, which are essential for germ cell maturation. This method eliminates the necessity for gonadotrophin stimulation. Consequently, xenotransplantation of ITT into the testicular parenchyma of other species emerges as a promising alternative for ITT grafting.

Moreover, donor age has been shown to influence the efficiency of testis xenografting in several studies. The study conducted by Abrishami et al. [54] in 2010 demonstrated notable outcomes in terms of graft recovery rate, graft weight, seminiferous tubule count, and successful spermatid generation when employing testicular xenografts from prepubertal and pubertal donors. These findings underscore the potential of utilizing immature donors instead of mature donors for testicular tissue xenografting. The authors assumed that the more advanced spermatogenesis occurred in the donor testis tissue, the worse the recovery rate in the graft. Therefore, the higher spermatogenic activity in adult testis tissue would lead to graft degeneration. In addition, adult testis tissue might be more sensitive to ischemic injury before re-vascularization, but the vascularization efficiency of testis tissue xenografting between immature and adult remains disputable.

### The impact of supplementation with hormones and other factors

Several studies have investigated the potential of supplementation with hormones or other factors to enhance graft outcomes, yielding varied and controversial results. For instance, in mice with infant rhesus monkeys ITT xenografts, the administration of exogenous human chorionic gonadotropin (hCG) improved the growth of xenografted ITT, resulting in complete spermatogenesis [57, 58]. However, supplementing human slow-frozen ITT xenografts with N-acetylcysteine (NAC) or testosterone did not demonstrate a clear impact on germ cell survival or inhibition of apoptosis in the grafted ITT [59]. Additionally, in prepubertal human testis grafts, the exogenous administration of a combination regimen of gonadotrophins, hCG, and follicle-stimulating hormone (FSH) failed to improve germ cell survival and differentiation [60]. Given the uncertain results from administering hormones and other factors post-transplantation, it is logical to consider the site of transplantation and the transplantation technique as more critical factors in improving graft outcomes.

### Monitoring viable cell fate in ITT transplantation

In a series of previous studies by Chen [13, 16, 61–64], bioluminescence imaging (BLI) was employed to track germ cells, ovarian tissues, and ITT across more than a decade. BLI allows for the tracking of tissue-specific luciferase expression in transgenic mice, facilitating the real-time observation of various biological processes, including signaling events and protein-protein interactions within transplanted tissues in vivo [65]. Moreover, BLI empowers researchers to conduct longitudinal studies, enabling the continuous monitoring of germ cell fate over an extended duration. This capability proves invaluable in the assessment of the long-term impacts of ITT and the potential restoration of fertility in pre-pubertal males as they progress towards sexual maturity. Consequently, BLI was introduced as a valuable tool for the evaluation of germ cells and ITT in pre-pubertal male mice.

### Biomaterial scaffolds applied for transplantation

As mentioned in the previous part of the paper, there are still obstacles to overcome in ITT transplantation strategies. It has been proven that tissue engineering holds great potential to enhance various aspects of ITT transplantation. For instance, tissue engineering can contribute to the development of suitable scaffolds that mimic the natural architecture of testicular tissue. These scaffolds can provide structural support and guide the formation of functional testicular tissue.

Biomaterial scaffolds can be made from various materials, including natural polymers, synthetic polymers, or a combination of both. Natural polymers commonly used in scaffold fabrication include collagen, alginate, fibrin, and hyaluronic acid which are derived from natural sources such as animals or plants. Synthetic polymers such as poly-L-lactic acid (PLLA), polyglycolic acid (PGA), and poly (lactic-co-glycolic acid) (PLGA) are also widely employed due to their controllable properties and degradation rates.

Currently, numerous research groups are focused on enhancing composite materials and fabrication processes to develop superior scaffolds. Prior to transplantation of the scaffold with an implantable tissue construct into an organism, it is essential to thoroughly evaluate several key factors, including biocompatibility, biodegradability, mechanical properties, scaffold architecture, and manufacturing technology [66]. First, any scaffold for tissue engineering must be biocompatible so that it allows tissues to integrate and grow within the scaffold structure. Second, scaffolds cannot last for a long time, hence, they must be biodegradable, and degradation should not produce toxins. Third, the mechanical properties of the scaffold should be identical to the anatomical site into which it is going to be implanted. Fourth, scaffolds need to maintain a high porosity to achieve effective vascularization. The architecture of scaffolds should have an interconnected pore structure which is of vital importance for the discharge of wastes from the scaffold. Finally, the scaffold manufacturing process should be cost effective, and the final product should be available immediately for clinical use.

**Fig. 2:**
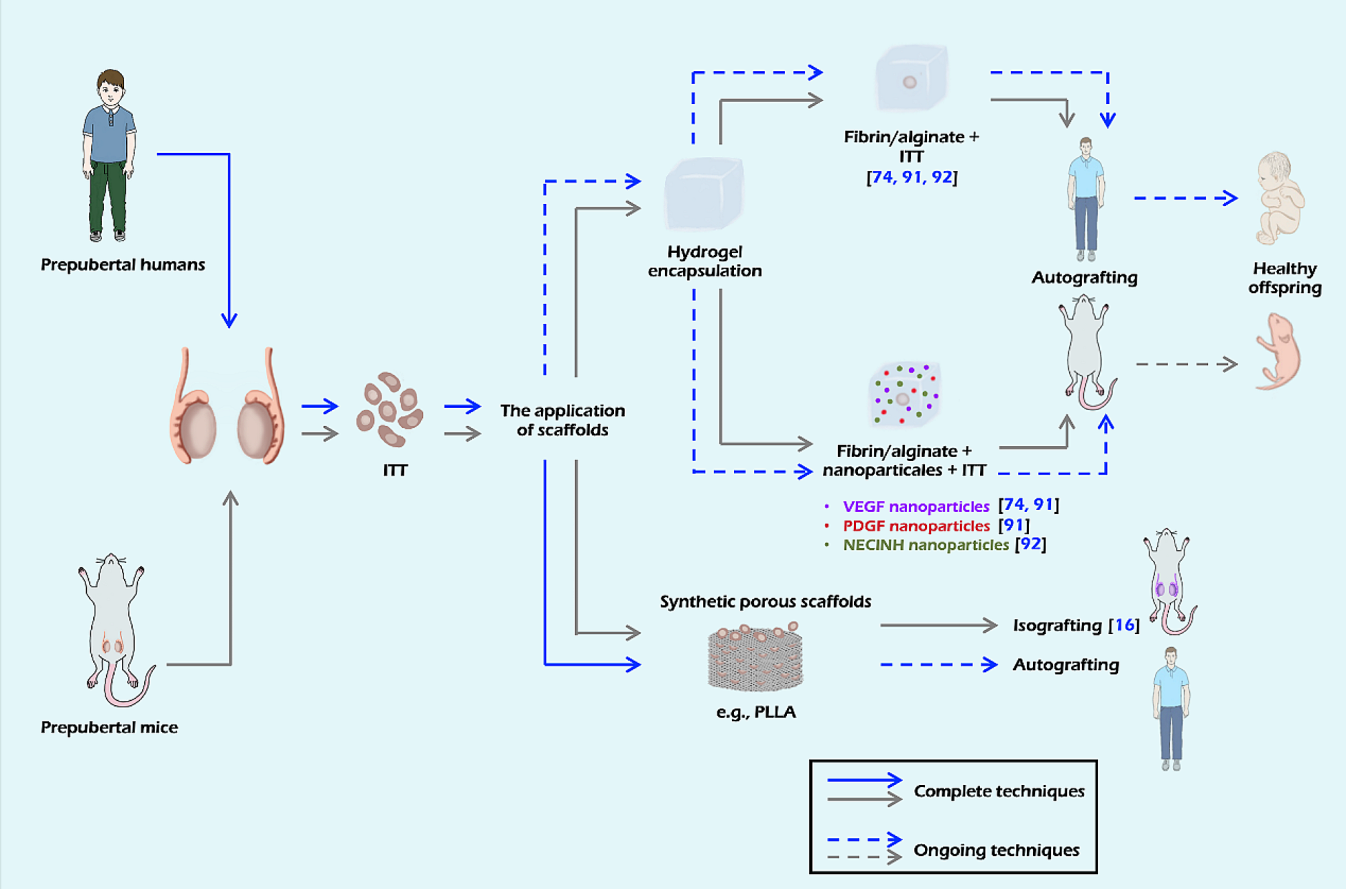
Schematic overview of two major scaffolding approaches for tissue engineering. The approaches include tissue fragment encapsulation in hydrogel matrix and premade synthetic porous scaffolds. Additionally, the figure highlights potential outcomes, such as the generation of offspring through ITT transplantation

Chan et al. summarized four scaffolding approaches over the past few decades [67]. In this paper, we aim to review two major scaffolding approaches for tissue engineering, including tissue fragment encapsulation in hydrogel matrix and premade synthetic porous scaffolds. We also discuss the evolution and prospects of ITT transplantation (Fig. 2).

### Hydrogels for tissue fragment encapsulation

Hydrogels are potential biomaterials for many medical applications, such as delivery systems for bioactive peptides and anticancer drugs [68, 69]. Hydrogels exhibit unique properties, notably their high water content, which is particularly beneficial for fragile or sensitive tissues that require a moist environment for optimal viability and function. In a study by Shojarazavi et al. [70] in 2021, they developed an innovative injectable hydrogel for cartilage tissue engineering by incorporating silk fibroin nanofibers into an alginate/cartilage extracellular matrix (ECM)-based formulation The study provided evidence that this specific hydrogel formulation holds promise in effectively repairing articular cartilage defects by successfully mimicking the natural cartilage environment. In addition, hydrogels can be designed with controlled porosity and permeability, which promote cell interactions by bidirectional diffusion of nutrients, oxygen, and waste [71, 72]. Overall, hydrogels provide an attractive platform for tissue fragment encapsulation due to their hydration capacity and permeability. These advantages make hydrogels an ideal candidate for tissue engineering and regenerative medicine.

Hydrogels can be classified into two categories based on their origin: natural or synthetic polymers. Natural polymers include elastin, collagen, gelatin, keratin, hyaluronic acid, chitosan, heparin, alginate, and fibrin. Synthetic polymers include polyethylene glycol (PEG), poly(N-isopropylacrylamide) (PNIPAAm), and polyacrylic acid (PAA). The main distinction between natural and synthetic hydrogels lies in their properties: natural hydrogels often exhibit excellent biocompatibility and bioactivity due to their resemblance to the native tissue environment, while synthetic hydrogels offer customizable properties and controlled degradation rates, making them suitable for specific applications. Taking natural polymers as an example, alginate is a notable anionic mucoadhesive polymer due to its terminal carboxyl groups [73]. Alginate offers several advantages for male fertility preservation. Poels et al. demonstrated its potential in cryopreservation by encapsulating ITT within alginate hydrogel, effectively improving tissue engraftment, and hence spermatogonial survival [74]. Alginate’s ability to mimic the extracellular matrices plays a pivotal role in supporting stemness potential during the cell cryopreservation process and initiating spermatogenesis following transplantation. Fibrin is another biomaterial used in countless surgical and endoscopic procedures such as improving the efficiency of hemostasis and wound healing [75]. Fibrin has been utilized not only as a matrix for cell growth and differentiation, but also as an excellent delivery system to achieve sustained drug release [76].

The selection of an appropriate hydrogel material that aligns with the specific requirements of the target tissue or cell type is crucial. The process of tissue encapsulation within hydrogels typically involves several key steps, including hydrogel selection, hydrogel preparation, tissue seeding, gelation or cross-linking, culture and maturation, and subsequent assessment of the viability, proliferation, and functionality of the encapsulated tissue within the hydrogel [18]. It is worth noting that the encapsulation process may necessitate optimization depending on the particular tissue type, hydrogel material, and intended application. Factors such as cell density, hydrogel concentration, gelation kinetics, and culture conditions must be carefully controlled to achieve the desired outcomes in tissue encapsulation.

### The application of premade synthetic porous scaffolds

Synthetic porous scaffolds are made by incorporating porogens in solid materials so that cells within the tissue can attach easily [77]. They can be designed with tunable properties to match the specific requirements of different tissues or applications. The scaffold’s pore size, porosity, and mechanical strength can be adjusted to create an optimal microenvironment for nutrient diffusion within the tissue. In addition, many synthetic porous scaffolds are designed to be biodegradable, meaning they can be gradually broken down and absorbed by the body as new tissue forms. Typically, the composition of porous scaffolds can be divided into three groups, ceramics, synthetic polymers and natural polymers. Synthetic polymers, such as polycaprolactone (PCL), PLLA, PLGA, or PEG, are commonly used due to their tunable properties and ability to support tissue development. Indeed, PLLA has been widely used as a biomaterial scaffold in the field of human reproduction due to its distinguished mechanical properties [61, 78]. A study conducted by Eslahi et al. [78] in 2013 investigated the application of a PLLA nanofiber scaffold on frozen-thawed neonatal mouse SSCs and testis tissues, resulting in a significant increase in the in vitro formation of neonatal spermatogonial cell clusters. It is noteworthy that while the use of PLLA nanofiber scaffolds may induce SSC differentiation rather than preserving clonogenic and proliferation potential during cell culture, it still demonstrates a substantial impact on germ cells in both clinical and tissue engineering applications.

Another study by Lu et al. [61] in 2022, used electrospun PLLA scaffolds in the transplantation of ITT from transgenic donors, to wild-type recipient mice (both 3-weeks-old) to evaluate ITT spermatogenesis compared with those without a PLLA scaffold. The main finding of the study demonstrated that the PLLA scaffold groups consisted of high-density sperm and a relatively high sperm count in comparison with control groups because of the similarity to normal natural testicular tissue ECM. This result reaffirmed that graft survival was enhanced and spermatogenesis in ITT was also improved by using a PLLA scaffold.

Additionally, it is crucial to take into account the size of PLLA scaffolds. Research conducted by Chen et al. highlighted that ITT from 4-week-old mice exhibited the highest potential for spermatogenesis when combined with fine PLLA electrospun scaffolds [16]. This favorable outcome can be attributed to the fact that the fibrous network structure of the fine PLLA electrospun scaffolds closely resembled the native structure of decellularized testicular albuginea, with fiber fineness measuring approximately 150 nm. This similarity not only mimics the natural microenvironment of the testicular tissue but also promotes high permeability, facilitating the timely delivery of blood flow, nutrients, and other essential biomolecules.

Beyond considering the physical properties of the initial PLLA scaffolds, the degradation behavior of PLLA plays a crucial role. As these polymers break down and are metabolized, they create vacant spaces within the tissue. These spaces serve as opportunities for regenerated cells and interstitial tissue to gradually fill, ultimately restoring structural integrity. In summary, degradable PLLA contributes to stabilizing the microenvironment through the process of recrystallization.

In summary, biomaterial scaffolds play a pivotal role in advancing innovative solutions, potentially leading to the development of artificial testis made by 3D printing of ECM. By employing 3D printing to fabricate biomaterial scaffolds using ECM-derived materials or by integrating ECM components into the scaffold, researchers can establish environments that closely mimic the natural testicular ECM. These meticulously engineered scaffolds can then be populated with testicular tissues. This comprehensive approach holds substantial promise for advancing our understanding of male reproductive biology and effectively addressing challenges associated with male fertility.

### Adjuvant agent: VEGF, PDGF, NECINH

Recently, researchers have continued to explore advanced techniques and strategies to enhance tissue encapsulation within hydrogels, such as incorporating bioactive molecules, and creating spatially patterned cell distributions. Tissue engineering can utilize bioactive factors to create a favorable microenvironment for transplanted ITTs. These factors can promote cell survival, proliferation, and differentiation, leading to improved graft integration and functionality. The survival of the transplants is influenced by the vascular connection between the graft and host. Sufficient blood supply results in cell survival through the availability of oxygen, along with abundant nutrients and factors. Therefore, during transplantation, the addition of adjuvant agents is employed to promote angiogenesis. Among these agents, vascular endothelial growth factor (VEGF), platelet-derived growth factor (PDGF), and necrosis inhibitor (NECINH) are commonly used. By encapsulating these factors within hydrogels, it becomes possible to regulate the release kinetics of the biomaterial factors. This controlled release mechanism enables sustained, localized, or targeted delivery of the factors to specific sites. Additionally, the hydrogel matrix serves to protect the factors from premature degradation, clearance, or rapid diffusion, thereby extending their presence at the desired target site.

VEGF is described as an angiogenic factor that induces endothelial cell proliferation and migration [79, 80]. Various studies have been conducted on the delivery of VEGF to promote angiogenesis, such as bone regeneration in femur defects [81], stimulation of wound healing in diabetic mice [82], and promotion of neovascularization in the infarcted heart [83]. Many studies have also evaluated the beneficial impact of the incorporation of VEGF microspheres into hydrogels, which promote the formation of blood vessels and facilitate the tissue healing process [84–86]. Sustained release of VEGF is therefore of critical importance for accelerating vascularization, and reducing the occurrence of poor endothelialization and incomplete vascularization [87]. Poels et al. aimed to improve spermatogonial recovery of ITT autografts in mice by encapsulating VEGF-nanoparticles (VEGF-NPs) in two types of hydrogels [74]. One was made of 1% alginate, and the other was made of fibrin (formed by fibrinogen and thrombin at a ratio of 1:1). The main finding in this study was that the viability of mouse spermatogonial germ cells was significantly improved (2-fold) as alginate hydrogel existed, regardless of VEGF-NP supplementation, compared to other grafted groups. This observation highlighted the superiority of alginate in promoting the viability of mouse spermatogonial germ cells. This could be explained by variations in structure between the two matrices. The 1% alginate used in this study had a pore size of 200 μm. In fact, pores over 100 μm in diameter were recommended for angiogenesis [88]. Furthermore, the antioxidant properties of alginate have been shown to mitigate the production of reactive oxygen species following hypoxia [89], thereby enhancing the survival of spermatogonia after transplantation.

However, newly generated blood vessels became leaky and prone to rupture [80]. Therefore, PDGF was added to the hydrogels in order to optimize angiogenesis. PDGF played an important role in the stabilization of neo-vascularization by modulating the proliferation and recruitment of perivascular cells [90]. In one study, Del Vento et al. observed that the graft vascular surface was significantly increased when combining VEGF-NPs with PDGF-nanoparticles (PDGF-NPs) after 21 days of transplantation. In addition, vascular maturity improved after 5 days of transplantation when PDGF was added simultaneously, which suggested that PDGF-NPs made revascularization in grafts occur more quickly [91].

Alginate hydrogel loaded with VEGF-NPs supported short-term angiogenesis due to short exposure to hypoxia, but this beneficial effect disappeared in the long run. Several studies revealed that localized delivery of a NECINH could be a potential candidate to improve ITT graft outcomes [91, 92]. Del Vento et al. selected NecroX-5™ to scavenge reactive oxygen and nitrogen species generated by mitochondria due to its significant protective effect against hypoxia during ITT transplantation procedures. The results showed that incorporation of testicular tissue into alginate hydrogels loaded with NECINH-nanoparticles (NECINH-NPs) markedly improved tissue integrity and spermatogonial survival in comparison with the alginate-encapsulated group, indicating the advantages of promising prepubertal fertility restoration with ITT transplantation. However, nanoparticles containing VEGF, PDGF and NECINH simultaneously did not show a positive effect on seminiferous tubule integrity. The interactions between these three molecules are open to dispute.

Advances in nanoparticles loaded with adjuvant agents provide a certain benefit to tissue engineering. Nanoparticles possess several advantageous features, including low toxicity, distinct drug properties (such as high drug loading capacity), potential for targeted delivery, tunable physicochemical characteristics, and precise control of behavior. These attributes make nanoparticles promising tools for enhancing the outcome of ITT transplantation [93–96].

## Conclusion

The development of male ITT preservation has made great progress by improving cryopreservation methods, transplantation strategies, and the application of tissue engineering. Both CSF and vitrification have been shown to be feasible ways to cryopreserve ITT. Successful offspring production has been achieved through vitrification and subsequent transplantation. Additionally, studies have revealed that transplanting larger ITT fragments, and transplanting into the scrotal site result in significantly higher rates of tissue survival. Moreover, the use of biomaterial scaffolds during graft transplantation has shown promise in enhancing graft survival and stimulating spermatogenesis. In conclusion, the cryopreservation and transplantation strategies discussed in this study, supported by tissue engineering approaches, represent significant advancements in the restoration of fertility for ITT. These strategies hold the potential to be translated to human ITT, thereby preserving fertility in prepubertal boys undergoing gonadotoxic therapies.

## Data Availability

Not applicable.

## Abbreviations

ITT: Immature testicular tissue
SSC: Spermatogonial stem cell
3D: Three-dimensional
CSF: Controlled slow freezing
NHP: Non-human primate
SSV: Solid surface vitrification
OPS: Open-pulled straw
GMP: Glass micropipette
CPA: Cryoprotective agent
DMSO: Dimethyl sulfoxide
EG: Ethylene glycol
PrOH: 1,2 – Propanediol
hCG: Human chorionic gonadotropin
NAC: N-acetylcysteine
PLLA: Poly L-lactic acid
PGA: Polyglycolic acid
PLGA: Poly (lactic-co-glycolic acid)
ECM: Extracellular matrix
PEG: Polyethylene glycol
PNIPAAm: Poly(N-isopropylacrylamide)
PAA: Polyacrylic acid
PCL: Polycaprolactone
VEGF: Vascular endothelial growth factor
PDGF: Platelet-derived growth factor
NECINH: Necrosis inhibitor
VEGF-NP: VEGF-nanoparticle
PDGF-NP: PDGF-nanoparticle
NECINH-NP: NECINH-nanoparticle
